# Magrolimab Therapy in Conjunction with Conventional Chemotherapeutics Slows Disease Progression in Pediatric Acute Myeloid Leukemia Patient-Derived Xenograft Models

**DOI:** 10.3390/cancers17091509

**Published:** 2025-04-29

**Authors:** Julia G. Kim, Sohani K. Sandhu, Ritesh V. Dontula, Josh J. Cooper, Jaden Sherman, Max Rochette, Rehan Siddiqui, Lana E. Kim, Michelle S. Redell, Alexandra M. Stevens

**Affiliations:** Department of Pediatric Hematology/Oncology, Texas Children’s Cancer Center, Baylor College of Medicine, Houston, TX 77030, USA

**Keywords:** CD47, AML, antibody-drug conjugate, immunotherapy, preclinical

## Abstract

**Background/Objectives:** Magrolimab (Magro) is a humanized naked anti-CD47 monoclonal antibody that blocks the SIRPα CD47 interaction, allowing macrophages to target and destroy cancer cells. To evaluate its preclinical efficacy in vivo, Magro was tested as a single agent and in combination with conventional chemotherapy drugs, Cytarabine (Ara-C) or Azacitidine (Aza), in three pediatric AML (pAML) patient-derived xenograft (PDX) models—AML006 (KMT2A::MLLT1), AML010 (+10, WT1), and AML013 (KMT2A::MLLT4). **Methods:** After PDX model establishment, mice were assigned to treatment groups hulgG4 (VC, vehicle control for Magro), Magro, Ara-C + VC, Aza + VC, Ara-C + Magro, and Aza + Magro, and then followed for survival. Mice that met humane euthanasia endpoints and at the culmination of experimental timelines had tissues harvested to measure disease burden. **Results:** Magro alone significantly improved survival in AML006 (*p* < 0.0001) and AML013 (*p* = 0.003) and decreased bone marrow (BM) disease burden in AML006 (*p* = 0.009) and AML013 (*p* = 0.002). Ara-C + Magro therapy led to significantly improved survival in all three models and significantly decreased BM disease burden in AML006 (*p* < 0.0001) and AML013 (*p* = 0.048). Aza + Magro therapy led to significantly improved survival in AML013 (*p* = 0.047) and AML010 (*p* = 0.017) and significantly lower BM disease burden in AML010 (*p* = 0.001). **Conclusions**: Interestingly, the two models that demonstrated improvement in survival with Magro harbored KMT2A rearrangements, suggesting a subset of patients that may be more responsive to the effects of CD47 blockade. As this drug is being evaluated for use in other malignancies, future studies may focus on investigating the importance of biomarker-based patient selection.

## Simple Summary

Magrolimab, an anti-CD47 monoclonal antibody, is a promising targeted agent for pediatric acute myeloid leukemia (pAML), a patient population with a high need for novel therapeutics to improve current poor clinical outcomes. The aim of our study was to assess the efficacy of Magrolimab in three pAML pre-clinical models in combination with the conventional chemotherapy drugs Azacitidine and Cytarabine. Our work showed that Magrolimab, as a single agent or in combination with Cytarabine, was effective in slowing disease progression in pAML pre-clinical models with specific genetic changes, suggesting potential efficacy in subsets of pAML patients. These results highlight the importance of testing novel agents across diverse subtypes of a heterogeneous disease such as AML.

## 1. Introduction

Pediatric acute myeloid leukemia (pAML) is the second most common type of leukemia in children, affecting seven out of every million children every year [1]. Despite advances in treatment, the prognosis for children with AML remains worse compared to acute lymphoblastic leukemia (ALL) [2]. Many children experience short- and long-term side effects from pAML treatment, and the improvements in 5-year overall survival (OS) achieved through better risk stratification, dose intensification, and refined supportive care have plateaued at around 60% [1,3]. Relapse rates remain high, with 35% of low-risk patients experiencing relapse, and a concerning OS of only 20% for those relapsing within 12 months [2,3]. These factors underscore the urgent need for preclinical and clinical research to identify more effective, targeted therapies specific for pAML.

Advances in genomic research have expanded our understanding of the genetic drivers of pAML. It is now clear that pAML is genetically distinct from adult AML. Key leukemogenic drivers in pAML include KMT2A rearrangements, mutations in RAS genes, FLT3, WT1, KIT, and NUP98 fusions [2,4]. By contrast, adult AML is often driven by mutations in FLT3 (especially FLT3-ITD), NPM1, DNMT3A, and IDH1/2 [5]. This divergence in genetic drivers suggests that therapies that are effective for adult AML may not translate to pAML. Given the rarity of pAML cases, it is essential that treatments moving forward to clinical trials have a strong likelihood of being effective in children with pAML’s unique genetic landscape [3].

Cluster of differentiation 47 (CD47) is a potential target for pAML. Normally expressed in cells as a “don’t eat me” signal, this surface protein inhibits programmed cell removal by the innate immune system [6]. While normal cells also express CD47, many cancer types, including AML, overexpress this receptor [7]. Importantly, CD47 overexpression in AML correlates with inferior EFS and OS. While patients with FLT3-ITD were identified as having higher expression of CD47, inferior survival associated with high CD47 expression remained even when FLT3-ITD patients were excluded from the analysis [8]. Targeting CD47 may promote phagocytic elimination of AML, making it a promising adjunctive target in pAML treatment.

Magrolimab (Magro, previously known as 5F9) is a monoclonal antibody that blocks CD47 from interacting with SIRPα receptor on macrophages and dendritic cells [6]. Preclinical studies in adult AML models have shown promising results for Magro monotherapy. For example, an in vitro study using AML leukemic stem cells (LSCs) co-cultured with macrophages demonstrated significant phagocytosis when exposed to anti-human CD47 (hCD47) antibodies, but not with anti-human CD45 (hCD45) antibodies or IgG1 control antibodies [8]. Additionally, in vivo studies showed Magro successfully eliminated AML in xenograft models: 16 out of 20 Magro-treated mice across three different xenograft models reached long-term disease-free survival [6]. AML cells from mice treated with Magro engrafted fewer secondary recipients, compared to cells from mice treated with control IgG [8]. These findings suggest that Magro could target the LSC component of the AML population.

While Magro monotherapy has shown promise, its efficacy has been enhanced when administered as combination therapy. In a bladder cancer xenograft model, Magro combined with gemcitabine-cisplatin inhibited tumor progression, an effect not observed with Magro or chemotherapy alone [9]. For hematologic malignancies, Magro with rituximab led to robust disease elimination in non-Hodgkin’s lymphoma xenograft models, whereas Magro monotherapy produced modest effects [6]. Similarly, Magro combined with Azacitidine (Aza) produced prolonged disease elimination and improved OS in xenograft models with the AML cell line HL-60 compared to either agent alone [10]. A potential mechanism for the enhanced effects of Magro combination therapy was identified by Rinaldi et al. They showed that Magro in conjunction with ruxolitinib increased the expression of the pro-phagocytic protein, calreticulin, in CD34+ bone marrow (BM) cells from myelofibrosis (MF) patients. This effect was significant compared to calreticulin expression from either drug alone. The effect of increased pro-phagocytic signal (calreticulin) aided by the suppression of the anti-phagocytic signal (CD47) provides a plausible biologic rationale leading to reduced MF cell survival after ruxolitinib + Magro therapy vs. either agent alone [11]. These findings support further investigation of Magro in combination with conventional chemotherapeutic agents.

Promising results from multiple early-phase trials with Magro had encouraged proceeding to higher-phase clinical trials involving Magro. Notably, in a phase 1b trial (NCT0324879), 44% of adults with high-risk AML and myelodysplastic syndrome (MDS) patients achieved complete remission with Magro + Aza [12]. The subsequent phase 2 trial (NCT04435691) found that 80% of newly diagnosed high-risk AML patients responded to the triple combination of Aza, Venetoclax (Ven), and Magro [13]. Another clinical trial focused on applying Magro to relapsed/refractory diffuse large B-cell lymphoma (DLBCL) and follicular lymphoma (NCT02953509). The phase 1b found that 30 mg/kg of Magro led to 100% CD47 receptor occupancy on circulating white and red blood cells, leading to objective and complete response rates (CRR) of 40% and 33% for DLBCL, and 71% and 43% in follicular lymphoma [14]. A phase 2 trial with indolent non-Hodgkin’s lymphoma (follicular and marginal zone lymphoma) using Magro and rituximab showed an objective response rate (ORR) of 50% and CRR of 36% [15]. A phase 2 trial focusing on DLBCL demonstrated a 24% ORR and 12% CRR [16]. A clinical trial of Magro for relapsed/refractory multiple myeloma (NCT04802446) found no dose-limiting toxicities for Magro combined with the standard-of-care regimen of daratumumab, pomalidomide, dexamethasone, bortezomib, and carfilzomib [17]. In this trial, some partial responses were observed in the multiple myeloma patients. Solid tumors early phase trials showed Magro to be well tolerated overall, with some encouraging early signs of efficacy for multiple malignancies: (NCT04854499) metastatic head and neck squamous cell carcinoma, (NCT04827576) multi-arm solid tumors involving metastatic non-small cell lung, urothelial, and metastatic small cell lung cancer, (NCT04958785) triple-negative breast cancer, and (NCT02953782) colorectal cancer [18,19,20,21].

Despite these encouraging early results, response rates for the phase III trials have been less encouraging. In the ENHANCE-2 trial (NCT04778397), Magro + Aza did not significantly improve outcomes compared to standard care in adult AML patients with TP53 mutations [22]. Furthermore, grade ≥ 3 adverse events (anemia, infections) were more commonly seen in the Magro + Aza group than in the control group. Similarly, the ENHANCE-3 trial (NCT05079230) reported an increased risk of infection and respiratory failure in patients receiving Magro + Ven + Aza [23]. For patients with MDS and AML, anemia-related toxicities were frequently observed, and while this side effect profile is less of an issue, clinical trial development of Magro has been halted for both hematologic and solid tumors at this time [24].

At the time this preclinical testing was conducted, the promising results from early phase clinical trials justified investigating the effects of Magro in pAML, which is genetically distinct from adult AML. In particular, the importance of biomarker-based application of Magro was emphasized in a longitudinal study following the NCT04435691 trial of high-risk AML patients treated with Aza, Ven and Magro. Based on the analysis of the BM and peripheral blood (PB) samples of 11 patients enrolled in the study at baseline and during treatment with single-cell RNA-seq and CyTOF, it was found that responders to the Aza + Ven + Magro treatment had decreased TP53 mutant clone burden. In addition, the responders had increased T cell activation and CD8+ T cell activation and reduced expansion of inflammatory myeloid subsets. These findings suggested that patienta’ genetic profile and the immune profile of the host impact the response to Magro therapy [25].

In the present study, we evaluated the efficacy of Magro as a single agent and in combination with traditional chemotherapy agents, Aza and Cytarabine (Ara-C), in three pAML patient-derived xenograft (PDX) models. The PDX models have different genetic fusions and mutations that are all frequently represented in pAML: AML006 (KMT2A::MLLT1), AML010 (+10, WT1), and AML013 (KMT2A:MLLT4). We assessed the effects of Magro through disease progression, OS, and disease burden at the time of euthanasia for the PDX models. Given the heterogeneous nature of pAML, this approach provides valuable insights into Magro’s therapeutic potential in genetically distinct pAML cases.

## 2. Materials and Methods

### 2.1. Patient-Derived Xenograft (PDX) Models

Viably cryopreserved pAML vials (>85% AML blasts) from established PDX models from the Texas Children’s Hospital/Baylor College of Medicine were selected for this study (Table 1). PDX models were established by injecting NSGS mice (aged 5–8 weeks) with 2 × 10^5^ AML cells/mouse following a previously described BCM Institutional Animal Care and Use Committee-approved protocol [26].

### 2.2. Magro and Chemotherapy Agents

Magro and vehicle control (VC for Magro, hulgG4) were provided by Gilead Pharmaceuticals (Foster City, CA, USA). Both agents were diluted with phosphate-buffered saline (VWR, Radnor, PA, USA) to the necessary treatment concentrations. Ara-C (98% purity, Sigma Aldrich, Burlington, MA, USA) and Aza (98% purity, VWR) were diluted using sterile normal saline (VWR, Radnore, PA, USA).

### 2.3. Treatment

After flow confirmation that the majority of mice had ≥1% hCD45+ human pAML blasts in murine PB, mice were allocated to treatment groups based on sex and blast percentage to ensure even distribution: VC, Magro, Ara-C + VC, Aza + VC, Ara-C + Magro, and Aza + Magro.

**Figure 1:**
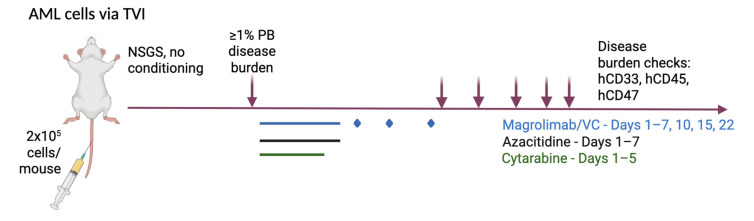
Schema of the treatment plan.

Magro and VC were injected in a dose escalation manner: 0.1 mg/kg/dose intraperitoneal (IP) days 1, 2, 3; 0.5 mg/kg/dose day 4; 1 mg/kg/dose days 5, 6; and 10 mg/kg/dose days 7, 10, 15, 22. Ara-C was given 50 mg/kg/day IP on days 1–5. Aza was given 2.5 mg/kg/day IP on days 1–7 (Figure 1).

Magro/VC dosing schedule for the study was optimized from a preliminary dosing test. Ara-C and Aza treatments were based on established dosing protocols.

### 2.4. Disease and Clinical Monitoring

Mice were consistently monitored for disease progression. Murine PB was analyzed by flow cytometry for human AML blasts (hCD33-AF700, hCD45-APC-Cy7, and hCD47-BV421 from Biolegend, San Diego, CA, USA) every 1–2 weeks after starting drug treatment. Samples were processed and run on the BD Symphony A5 following established flow cytometry protocols [26].

We followed two criteria for determining humane endpoints: ≥40% hCD45+ in murine PB detected by routine flow cytometry, or clinical signs such as loss of 20% of their initial body weight, labored breathing, hunched posture, or tumors > 1 cm.

### 2.5. Humane Euthanasia and Tissue Collection

Mice fulfilling humane endpoint criteria from above were euthanized following established protocols [26]. Tissues—PB, spleen (SP), and BM—were harvested and analyzed by flow cytometry for disease burden. All AML010 mice were euthanized for clinical illness by day 43, but the experiment was terminated for AML013, AML006, and AML006 Aza replicate mice on days 152–153, 70–72, and 73–75, respectively (Appendix A, Table A1).

### 2.6. Data Analysis Methods

Statistical significance in OS between different treatment groups was assessed by the log-rank test of the Kaplan–Meier survival data. The percentages of +hCD45 and +hCD47 cells in murine PB and tissues from euthanized mice were compared between treatment groups using a non-paired *t*-test. Statistical analyses were performed using GraphPad Prism (v 9.1.1) from GraphPad Software Inc.

## 3. Results

### 3.1. Tracking Survival Across Treatments and Models

**Figure 2:**
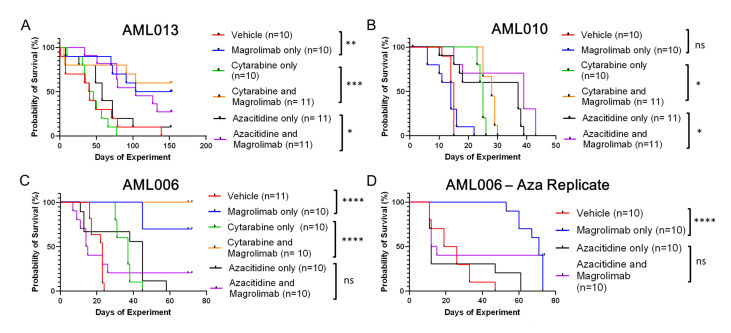
Kaplan–Meier graphs showing the probability of survival of the mice across models and treatment groups ((**A**)—AML013 all treatment groups, (**B**)—AML010 all treatment groups, (**C**)—AML006 all treatment groups, and (**D**)—AML006 Aza Replicate), with Day 0 counting as the first day of treatment. Mice that were euthanized due to end of experimentation were censored from the data. n indicates the number of mice included in each treatment group (* *p* ≤ 0.05, ** *p* ≤ 0.01, *** *p* ≤ 0.001, **** *p* ≤ 0.0001) (GraphPad).

Overall, Magro alone and in combination with Ara-C and Aza statistically improved survival to variable extents across all three pAML models. The administration of Magro alone resulted in significant improvement in median OS in two of three models: AML006 (first cohort): 23 vs. undefined days (*p* < 0.0001); AML006 (second cohort): 23 vs. undefined days (*p* < 0.0001); and AML013: 41 vs. 129 days (*p* = 0.01). Median OS for AML010 was 14 vs. 15 days (*p* = ns) (Figure 2).

All three models tested had improved OS for Ara-C + Magro compared to Ara-C + VC. AML006 and AML010 had significantly prolonged survival when mice were given combination therapy of Ara-C + Magro. When comparing the median survival, half of AML006 Ara-C + VC mice succumbed to disease by day 37, while the median survival for Ara-C + Magro was undefined, since all 10 were still alive at day 73 of the experiment (*p* < 0.0001). Similarly, the median survival for AML013 Ara-C + VC mice was day 43, while the median survival for Ara-C + Magro was also undefined, since six mice survived until day 152 of the experiment (*p* = 0.001) (Table A1). The median survival for AML010 Ara-C + VC mice was day 25, while the median survival of Ara-C + Magro mice was day 28 (*p* = 0.002). While the data was statistically significant for AML010, there was little clinical distinction in the survival differences between the two treatment groups.

**Figure A1:**
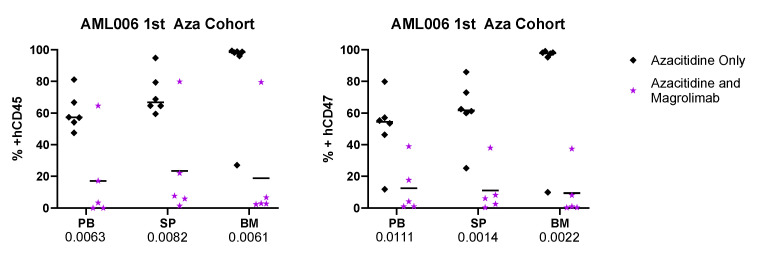
Percentages of +hCD47 and +hCD45 positivity in mice peripheral blood, spleen, and bone marrow at the time of harvest for the six Aza only and five Aza + Magro mice that had data available in the first cohort of AML006. Data points demonstrate that five mice had minimal disease burden at the time of harvest.

For assessing Magro in combination with Aza, two of three models had significantly improved OS from the Aza + Magro combination therapy when compared to Aza + VC. The median OS for AML013 for Aza + VC mice was day 58, while the median OS for Aza + Magro mice was day 104 (*p* = 0.047). AML010 Aza + VC had a median survival to day 37, while Aza + Magro mice had median survival to day 39 (*p* = 0.017, though this difference in survival was not felt to be clinically meaningful). Median OS for AML006 for Aza + VC vs. Aza + Magro was 15 vs. 45 days (*p* = ns due to intersection of survival curves, first cohort) and 14 vs. 12 days (*p* = ns, second cohort). For the initial cohort of AML006 mice tested, several mice receiving Aza became clinically ill and were found to have minimal leukemia burden (Figure A1). Because of this unexpected toxicity, the cohort was repeated (Appendix A). The second treated cohort yielded survival to day 73 in four of the Aza + Magro-treated mice, though the difference between the two groups was also not significant, as described above.

**Figure A2:**
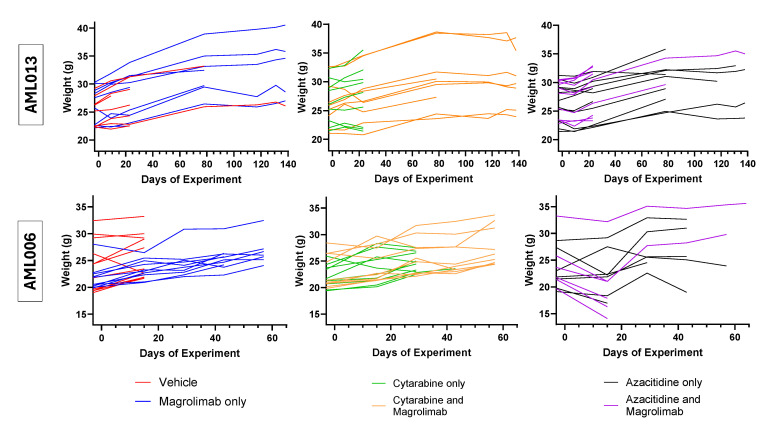
Body weights of AML013 mice and AML006 mice throughout the experiment, split up in the following groups: VC vs. Magro, Cytarabine + VC vs. Cytarabine vs. Magro, and Azacitidine + VC vs. Azacitidine + Magro. AML010 mice body weights are not available due to inconsistent weight collection for this cohort.

Tracking body weights of the mice in the AML006 and AML013 cohorts throughout the experiment demonstrated no significant differences between the treatment groups, indicating the tolerance of the drugs individually and in combination (Figure A2).

### 3.2. Tracking Disease Burden and CD47 Expression

To supplement the OS data from the Kaplan–Meier analyses, we tracked the progression of pAML in xenograft models by monitoring hCD45 positivity in PB. hCD47 expression was also assessed to evaluate Magro’s ability to block CD47 receptors on pAML cells, as high hCD47 levels suggest resistance to phagocytosis by macrophages. Mice treated with Magro generally showed delayed disease progression and reduced hCD45 levels, indicating slower proliferation of pAML cells. Additionally, Magro treatment reduced the surface expression of hCD47, demonstrating it successfully blocked hCD47 by preventing flow antibody from binding to those cells.

**Figure 3:**
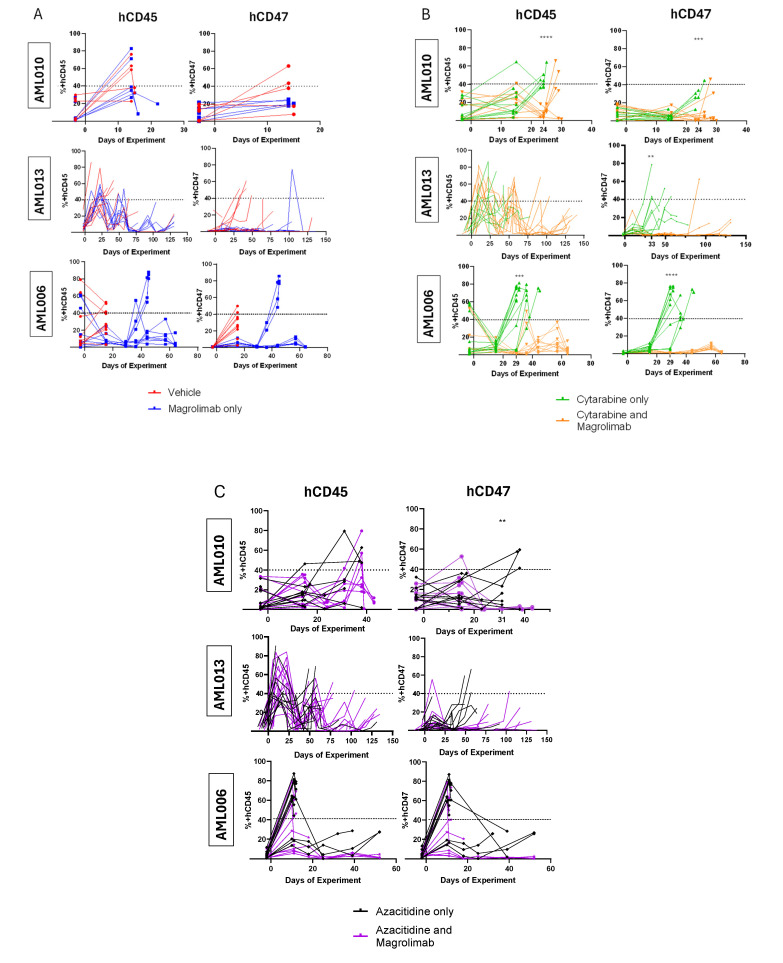
Detection of % of cells positive for hCD45 and hCD47 in murine peripheral blood throughout the course of experiment for AML006 (replicate data), AML010, and AML013. Figures compare the effects of: (**A**) vehicle control (VC) vs. Magrolimab (Magro), (**B**) Cytarabine (Ara-C) + VC vs. Ara-C + Magro, and (**C**) Azacitidine (Aza) + VC vs. Aza + Magro. Days of experiment are counted with day 1 being the first day the mice were given treatment with intraperitoneal injection. The dashed horizontal line at 40% for each graph marks the disease burden limit for humane euthanasia in this experiment. (** *p* ≤ 0.01, *** *p* ≤ 0.001, **** *p* ≤ 0.0001) (GraphPad).

In cases where Magro monotherapy improved OS, tracking hCD45 positivity revealed that this benefit stemmed from maintaining PB hCD45 levels below 40%, the threshold for humane euthanasia (Figure 3A). For AML010 (Figure 2), though there was no significant improvement in OS for Magro-treated mice compared with VC (Human IgG), 2/10 Magro-treated mice maintained PB hCD45 below 40% until the conclusion of the experiment (Figure 3A). Importantly, in all three PDX models (basal hCD47+ 92–98%, Table 1), Magro successfully blocked hCD47 surface expression. For AML010, hCD47 levels were close to 20% on day 14–15, while hCD45 levels ranged from 20 to 80%. For AML013, hCD47 levels were negligible throughout the majority of the experiment despite measurable AML disease burden. Similarly, for AML006, the hCD47 levels were also less than hCD45 levels in nearly all mice throughout the experiment (Figure 3A).

Ara-C and Magro combination treatment prolonged survival and slowed disease progression for all three xenograft models compared with Ara-C-only treatment. For AML010 and AML006, PB hCD45 levels were significantly higher in the Ara-C-only group than the Ara-C + Magro group at key timepoints before the majority of the mice succumbed to disease (AML010 on day 24, *p* < 0.0001; AML006 on day 29, *p* = 0.0001; Figure 3B). Although early timepoint differences were not observed for AML013, Ara-C + Magro-treated mice lived significantly longer, with hCD45 levels remaining below 40% for over 49 days after the last Ara-C + VC mouse died. hCD47 expression was significantly reduced in the combination therapy group for all three models at their respective timepoints (AML010 on day 24, *p* = 0.0001; AML013 on day 33, *p* = 0.007; and AML006 on day 29, *p* < 0.001). In AML013, hCD47 levels remained under 5% until day 76, despite increasing hCD45 levels, suggesting a prolonged Magro effect. Similar trends were observed in AML006, where hCD47 levels remained negligible until the end of the experiment. These data demonstrate that Ara-C + Magro improved survival via slowing of disease progression and effectively blocked hCD47 for all three PDX models. Notably, for the AML006 and AML013 models, the survival advantage was most clinically meaningful.

In contrast, the Aza + Magro combination yielded variable results compared with Aza + VC. For AM010, there was no significant difference in hCD45 levels between Aza + Magro vs. Aza + VC (Figure 3C), consistent with a lack of significant difference in median survival (Figure 2). This was despite the substantial hCD47 blockade in this model (hCD47 levels were significantly lower for Aza + Magro-treated mice at day 31, *p* = 0.029). For AML006 s cohort, the hCD45 and hCD47 levels were lower than 10% after day 10 throughout the course of the experiment for Aza + Magro, but Aza + VC was sufficient for prolonged disease control, and the addition of Magro did not enhance the activity of Aza in this model or improve OS (Figure 2). In contrast, for AML013, Aza + Magro treatment resulted in both strong hCD47 blockade and delayed disease progression, with hCD45 levels remaining below 40% after day 100 and associated improved OS in mice receiving Aza + Magro when compared to Aza + VC (Figure 2).

### 3.3. Disease Burden at Harvest

hCD45 percentages in PB, SP, and BM tissues at harvest showed how the disease manifested in the xenograft models. Ideally, effective treatment will result in the eradication of pAML cells from all tissues, including BM, the niche for LSCs. However, the assessment of disease burden in both the BM and in extramedullary sites, such as the spleen, can be informative to assess efficacy patterns and whether the reduction in PB and SP disease burden correlated with BM disease burden or if they were independent.

**Figure 4:**
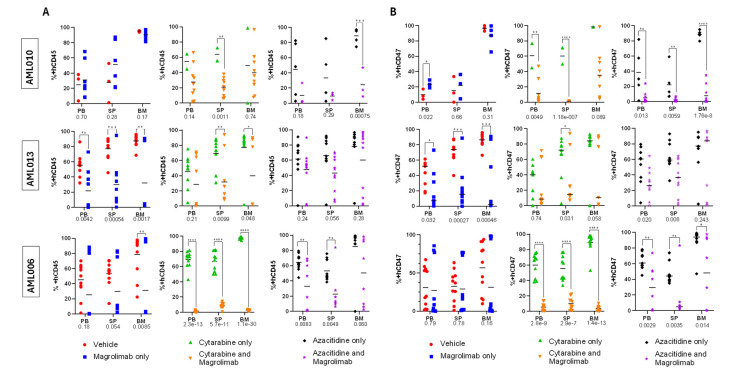
Percentages of +hCD45 (**A**) and +hCD47 (**B**) in mice peripheral blood (PB), spleen (SP), and bone marrow (BM) at the time of harvest for AML006 (n = 2–5), AML010 (n = 9–11), and AML013 (n = 8–10). (* *p* ≤ 0.05, ** *p* ≤ 0.01, *** *p* ≤ 0.001, **** *p* ≤ 0.0001) (GraphPad).

Magro monotherapy resulted in a significantly lower BM disease burden in AML013 and AML006 xenograft models (Figure 4A). For AML013, the levels of hCD45 and hCD47 were significantly lower in Magro-treated mice vs. VC in all three tissue types (Figure 4A,B). AML006 Magro-treated mice had statistically lower hCD45 in the BM only. Notably, there was disease burden variability in Magro-treated mice at the time of tissue harvest with three mice with substantial disease burden at harvest. These results correlate well with the data from the spaghetti plots (Figure 3A), showing that Magro alone resulted in prolonged disease control for the remaining treated mice (7 out of n = 10). Additionally, AML006 hCD47 levels did not differ significantly between VC and Magro-treated mice, implying that Magro did not effectively block hCD47 and, therefore, likely would not have induced greater phagocytic removal for this xenograft model (Figure 4B). This may be related to the longer survival of many of the mice in this cohort.

Ara-C + Magro significantly lowered BM disease burden for AML013 and AML006 (Figure 4A). The disease burden in the spleen of Magro-treated mice was substantially lower for all three xenograft models (Figure 4A). Furthermore, combination therapy decreased hCD47 detection in all three tissues for AML006 (Figure 4B). Peripheral tissues in AML013 and AML010 (SP only for AML013 and both PB and SP for AML010) demonstrated a significant decrease in hCD47 (Figure 4B).

Aza + Magro significantly lowered the disease burden in BM at harvest in AML010, but not in AML006 or AML013. Though this combination did not suppress AML006 in the main tissue of interest, it demonstrated lower hCD45 disease burden in both peripheral tissues (Figure 4A). For both AML006 and AML010 models, there was a significant decrease in hCD47 detection for all tissue types at harvest (Figure 4B).

## 4. Discussion

This study examined the effect of Magro monotherapy and Magro in combination with Aza and Ara-C for three pAML PDX models, AML010 (WT1), AML013 (KMT2A::MLLT4), and AML006 (KMT2A:MLLT1). Major differences in the genetic drivers of adult AML vs. pAML and a gap in targeted chemotherapy for pAML have established the importance of robust preclinical testing of all novel therapies being considered for incorporation into pAML treatment. Promising preclinical results for Magro monotherapy and combination therapy for adult AML, along with encouraging Phase 1 and 2 studies for AML and MDS, supported the need to execute well-designed preclinical studies of Magro for pAML. We found that in pAML PDX models, Magro monotherapy was generally effective in improving OS and maintaining low PB disease burden, and it resulted in the most effective disease suppression when administered with Ara-C.

Azacitidine is a drug of interest in pAML because it is well tolerated and thus can be easily combined with novel agents without exceeding the limits of tolerability of some more traditional pAML chemotherapeutic regimens. Moreover, the hypomethylation activity of Aza increases the expression of calreticulin and CD47 on cancer cells, which increases both the pro-phagocytic and anti-phagocytic signals on the cells [10]. For this reason, we hypothesized that there would be synergy through combining Aza and Magro. Cytarabine is the most important cytotoxic chemotherapeutic for pAML, making this another important agent for combinatory studies with Magro.

Overall, two models in our study—AML006 and AML013—had anti-leukemic benefits from Magro monotherapy. In both models, Magro treatment resulted in longer OS, reduced PB hCD45, and hCD47 below 40% for most of the course of the experiment, and significantly lower hCD45 levels in the BM at the time of harvest. In contrast, AML010 did not demonstrate significant improvement from Magro monotherapy.

The combination of Ara-C with Magro led to a significant increase in survival in all three models and a significant decrease in BM disease burden in AML006 and AML013. Additionally, PB hCD45 levels were significantly lower in Ara-C + Magro-treated mice for AML010 at day 24 and AML006 at day 29. The significantly lower levels of PB hCD47 during the experiment for all three models correspond to significantly lower hCD47 levels in tissues at harvest (AML010: PB and SP, AML013: SP, AML006: all three). Collectively, this preclinical data support the efficacy of Magro + Ara-C combination across all models.

Aza + Magro showed variable efficacy across the three models. Only AML010 and AML013 exhibited significantly improved OS, with AML013 showing a notable decrease in PB hCD45 level to support the OS data. Although AML010 did not demonstrate a significant decrease in PB hCD45 levels, it did exhibit significantly lower disease burden in BM at harvest, further corroborating the OS data. Interestingly, there were significantly lower hCD47 levels in all three tissues for AML010, indicating successful binding of Magro to its target. AML006 showed no significant difference in OS and BM disease burden at harvest, although it did demonstrate modest activity of Aza + Magro, evidenced by lower levels of hCD45 in PB and SP, and significantly lower levels of hCD47 in all three tissues at harvest.

Despite these promising preclinical results, due to the lack of improved survival in adult trials of Magro in hematologic malignancies, Magro clinical trials at this time have been halted. As a result, further pre-clinical investigation of this drug for hematologic malignancies such as pAML is not being prioritized. Regardless, our study emphasizes the importance of biomarker-based patient selection when using novel drugs, as the models that worked best with Magro—AML013 (KMT2A::MLLT4) and AML006 (KMT2A::MLLT1)—both harbor a KMT2A rearrangement which, depending on the fusion partner, can be a high-risk genetic fusion. In comparison, AML010 (+10, WT1) demonstrated minimal clinical benefit from Magro monotherapy and in combination with Aza. This follows a retrospective study examining possible biologic rationale for the increased incidence of death in AML patients treated with Magro + Ven + Aza on the ENHANCE-3 trial (NCT05079230): data from sequencing of participants bone marrow samples revealed a higher incidence of death in patients with TET2 mutations [27]. The subgroup with TET2 mutations had higher incidence of death due to pneumonia or lung disease, which was theorized to be secondary to a maladapted innate response to infection, as TET2 mutated mature myeloid cells have pro-inflammatory features. Our data supports that biomarker-based selection of patients in future clinical trials has the potential to reduce drug toxicity reactions and improve chemotherapy outcomes.

LSCs are also an important focus of AML research, as this subgroup of AML cells is thought to be therapy-resistant and contribute to relapse [28]. Following the discovery of upregulation of CD47 receptors on LSCs [29] and the successful elimination of AML LSCs in preclinical studies using Magro [8], clinical trials have also suggested effects of Magro on LSCs. When assessing the BM samples of patients enrolled in the phase 1b trial (NCT03248479: 22 patients with AML and 47 with HR-MDS), proteomics found that Magro + Aza not only reduces leukemic blasts, but also impacts the bone marrow microenvironment by enhancing T cell infiltration [30]. This suggests that Magro may have relevant impacts on LSCs, which reside in protected BM niches. Given the importance of developing drugs with efficacy against LSC populations, evaluating Magro + chemotherapy’s effect on LSCs through serial transplantation and limiting dilution assays would be interesting.

## 5. Conclusions

Patients with pAML tend to have inferior prognoses, with an OS rate of 60% that sharply decreases to 20% for children who relapse [2,3]. We investigated the efficacy of a promising new agent, Magro, against pAML as a single agent and in combination with traditional back-bone chemotherapy agents, Ara-C and Aza, in three PDX models. We found that Magro monotherapy was effective against the two models in our study that had KMT2A rearrangements. The combination of Ara-C with Magro was active across all three models tested. Investigating the effect of Magro on LSCs would be an interesting next step, as it is important to develop drugs that will prevent relapses by targeting LSCs. Though clinical development of Magro for hematologic malignancies is currently paused, this study has contributed important information to the preclinical drug testing space. This work demonstrates the importance of preclinical testing to assist in biomarker-based selection of patients for novel therapies, as PDX models with KMT2A rearrangements seemed to benefit more from Magro treatment.

## Figures and Tables

**Table 1:** Characteristics of the 3 PDX models utilized—patient age at diagnosis (years), race and ethnicity, clinical timepoint used for PDX generation, risk group, clinical outcome, key mutations, and surface expression of CD47 shown. Additional data for these models are available on our publicly available PDX portal (https://pdxportal.research.bcm.edu/, accessed on 13 March 2025). Definitions: M: male, W: white, B: black, NH: non-Hispanic, DD: death due to disease, MRD: minimal residual disease.

Public Model Name	Age/ Sex	Race/ Ethnicity	Timepoint	Risk Group/ Clinical Outcome	Major Cytogenetic or Molecular Features	% CD47+
AML006	9/M	B/NH	Relapse	High risk (fusion); DD	KMT2A::MLLT1, KRAS, SETD2, WT1	98.40%
AML010	2/M	W/NH	Diagnosis	Low risk (MRD negative, non-prognostic genetics); alive	+10, WT1	95.10%
AML013	13/M	B/NH	Diagnosis	High risk (fusion); DD	KMT2A::MLLT4, BRAF, BCOR	92.80%

## Data Availability

Data may be obtained by emailing amsteven@texaschildrens.org.

## Abbreviations

The following abbreviations are used in this manuscript:

pAML	Pediatric acute myeloid leukemia
ALL	Acute lymphoblastic leukemia
OS	Overall survival
CD47	Cluster of differentiation 47
hCD47	Human cluster of differentiation 47
Magro	Magrolimab
DLBCL	Diffuse large B-cell lymphoma
CRR	Complete response rate
ORR	Objective response rate
LSCs	Leukemic stem cells
MF	Myelofibrosis
CD34	Cluster of differentiation 34
Aza	Azacitidine
MDS	Myelodysplastic syndromes
Ven	Venetoclax
Ara-C	Cytarabine
PDX	Patient-derived xenograft
VC	Vehicle control
PB	Peripheral blood
IP	Intraperitoneal
SP	Spleen
BM	Bone marrow
hCD45	Human cluster of differentiation 45

## Appendix A

### Explanation for Repeating Azacitidine + Magro Arms for AML006

Six mice (five harvested and one found dead) in our first AML006 trial died without evidence of high disease burden across both the Aza and Aza + Magro groups. Since this was not an issue in any prior cohorts of mice, it was likely not due to drug toxicity. Autopsy did not demonstrate GI issues, hemorrhages, or lesions, so no clear etiology of the early deaths was identified. As a result, we attribute the deaths to unexpected toxicity. Therefore, we repeated this arm of the experiment in a new cohort of mice and performed a second AML006 trial with just the Magro, VC, Aza, and Aza + Magro groups.

**Table A1:** Number of mice harvested for each experiment group when the experiment was terminated after establishing significance in OS data. NA: not applicable.

**Treatment Groups**	**AML010**	**AML013**	**AML006**	**AML006 (Aza Replicate)**
Date of experiment	NA	152–153	70–72	73–75
VC	0	1	0	0
Magrolimab	0	5	6	6
Ara-C + VC	0	0	0	NA
Ara-C + Magrolimab	0	6	10	NA
Aza + VC	0	1	0	0
Aza + Magrolimab	0	3	2	4
